# A cruise control for Parkinson's disease

**DOI:** 10.1186/1471-2202-12-S1-P304

**Published:** 2011-07-18

**Authors:** Steven J Schiff, Patrick Gorzelic, Alok Sinha

**Affiliations:** 1 Center for Neural Engineering, Penn State University, Pennsylvania, 16802, USA; 2 Department of Mechanical Engineering, Penn State University, Pennsylvania, 16802, USA

## 

As our knowledge of Parkinson's disease increases, we are developing sophisticated computational models of the neuronal networks that go awry in this condition. Such models can form a test bed for the rational design of control strategies to reduce the pathological dynamics. Although deep brain stimulation is becoming increasingly popular for treating Parkinson's disease, all of the present stimulation scenarios involve open-loop stimulation that does not take into account the brain's dynamics through feedback.

Most engineering control systems, such as automobile cruise controls, make use of variants of proportional-integral-differential (PID) control strategies in response to a measured quantity. We here design an optimized PID controller for Parkinson's disease modulation.

We adapted the sparse-structured model of a small network of synaptically coupled neurons in several basal ganglia areas, originally described by [4], to an efficient structured Matlab implementation. The major outputs from the basal ganglia are inhibitory signals from the globus pallidus interna (GPi) to its localized target areas within thalamus. Correspondingly, a natural view is that parkinsonian basal ganglia activity is transduced into motor symptoms through effects on thalamic dynamics. Specifically, computational [2] and data-based [1] analysis shows that pathological GPi synaptic outputs can compromise thalamic relay of excitatory signals, as can be assessed by calculation of a reliability index or by estimation of GPi output patterns.

We assume an overarching principle for control in Parkinson's disease – we never want to put more than 1 multicontact electrode shaft in the brain. We start with GPi as a target, since the clinical differences between GPi and STN stimulation effects have not been well documented in controlled studies, and the GPi is a larger and easier target into which to insert stereotactic electrodes (see discussion in [3]). We set up 2 empirical control schemes: 1) a *reliability control* based upon the fraction of the sensorimotor spikes, accurately relayed 1-for-1 by the thalamic cells, and a GPi *synaptic control* based upon the estimated effective output of the GPi.

We contrast several proportional control schemes based upon reliability: a frequency-proportional case, and a frequency-proportional-biased case. Although the energy expenditure is almost identical for both cases, the reliability is higher with adding a bias term.

We contrast several proportional control schemes using a GPi output controller. Using an amplitude-proportional-derivative control scheme, where the error is equal to the instantaneous calculated GPi synaptic output minus the average GPi output. We flip the sign of the derivative term, from positive to negative, to explore whether amplifying or suppressing the instantaneous response to the differential error is best. Use of a negative differential coefficient yields substantially greater reliability and lower energy expenditure.

**Figure 1:**
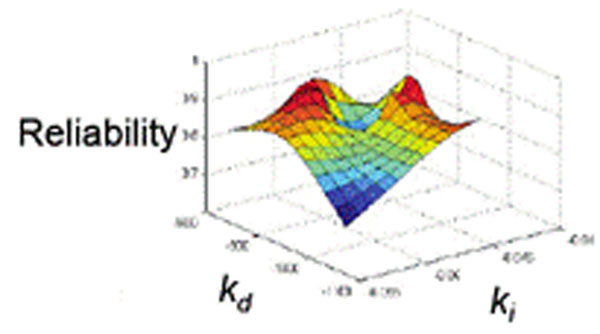
Thalamic reliability as a function of derivative, kd, and integral, ki, terms following exhaustive optimization.

Finally, we exhaustively optimize a PID controller using proportional, derivative, and integral terms, as shown in as shown in Figure 1.

We have shown the feasibility of rational PID controller design for Parkinson's disease. Both reliability, and GPi synaptic outputs, can be estimated from reduced model based sensors, and the results of the PID scheme instituted in trials with real feedback controllers. Developing these reduced models for incorporation into future devices is the next step of this research.
